# Coexisting Congenital Left and Right Coronary Artery Anomalies: A Rare Cause of Pediatric Cardiac Arrest

**DOI:** 10.1016/j.jaccas.2025.105290

**Published:** 2025-12-08

**Authors:** Samir Bhatia, Matthew Liava'a, Julian Ayer, Claire M. Lawley

**Affiliations:** a The Heart Centre for Children, Sydney Children's Hospitals Network, Westmead, New South Wales, Australia; b Faculty of Medicine and Health, The University of Sydney, Westmead, New South Wales, Australia

**Keywords:** coronary circulation, coronary vessel anomaly, ventricular fibrillation

## Abstract

**Background:**

Congenital left main coronary artery atresia (LMCAA) is an extremely rare anomaly associated with a high risk of myocardial ischemia and sudden cardiac arrest.

**Case Summary:**

We present the case of a 12-year-old boy who experienced a ventricular fibrillation arrest during exercise, primarily attributed to LMCAA with an anomalous right coronary artery (RCA) originating from the left aortic sinus considered as the potential contributing factor. Multimodal imaging was critical in diagnosis and surgical planning. The patient underwent successful surgical revascularization with left internal mammary artery grafting to the left anterior descending artery and unroofing of the anomalous RCA.

**Discussion:**

In children presenting with out of hospital cardiac arrest, accurate imaging for coronary artery anomalies remains paramount. The diagnosis of congenital LMCAA with an anomalous RCA (with high-risk features) represents a rare but potentially treatable cause.

**Take-Home Message:**

The case underscores the importance of early recognition and intervention in rare congenital coronary artery anomalies.

## Graphical Abstract

**Figure:**
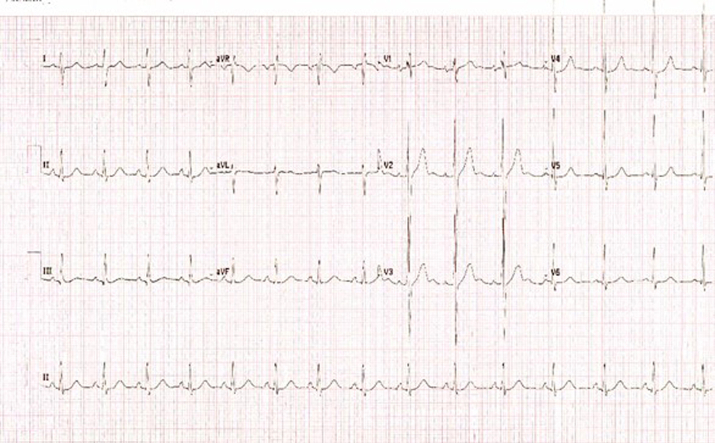

## History of Presentation

A previously healthy 12-year-old boy experienced cardiac arrest after soccer practice, with preceding dizziness and exertional fatigue. He had completed playing and lay down on the side of the field, he felt unwell, and subsequently while being carried to the car became unresponsive. Bystander cardiopulmonary resuscitation was initiated, and an automated external defibrillator was applied, which discharged. There was return of spontaneous circulation. He required intubation for pulmonary edema and was taken by ambulance to the quaternary children's hospital emergency department. After stabilization, the patient was transferred to the pediatric intensive care unit.

**Take-Home Messages**

• In children presenting with out of hospital cardiac arrest, accurate imaging for coronary artery anomalies remains paramount.
• The diagnosis of congenital LMCAA with an anomalous RCA (with high-risk features) represents a rare but potentially treatable cause.
• Multimodal imaging, including echocardiography, CT angiography, and cardiac catheterization may be considered; in some situations, stress imaging is recommended.
• Long-term follow-up after surgical intervention for anomalous coronary artery anatomy should be considered, to monitor graft patency, cardiac function, and assess for any inducible ischemia.

## Medical History

The patient had no known cardiac history but a history of mild exertional asthma. There was no family history of sudden cardiac death or heritable cardiac conditions.

## Differential Diagnosis

The differential diagnosis for a postexertional collapse in a shockable rhythm in an otherwise largely well child includes structural cardiac disease, cardiomyopathy, coronary artery pathology (both congenital and acquired, such as post-Kawasaki disease), primary ion channel disease (those typically presenting during exertion include catecholaminergic polymorphic ventricular tachycardia and type 1 long QT syndrome), Wolff-Parkinson-White syndrome, and other rare causes.^1^

## Investigations

**Figure 1:**
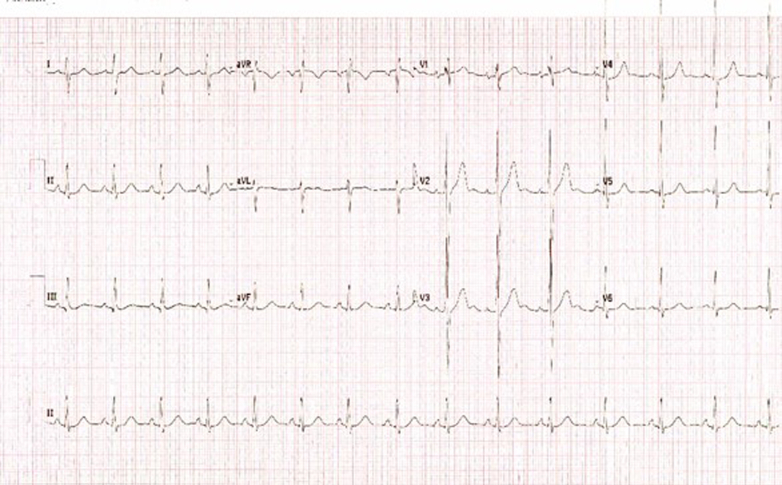
Initial Electrocardiogram Initial electrocardiogram demonstrating sinus conducted rhythm, QRS axis 98°, normal duration 89 milliseconds, T-wave upright from V_1_, giant T-wave V_2_, normal QTc interval, and no significant ST-segment change.

The initial electrocardiogram (ECG) demonstrated sinus conducted rhythm, QRS axis 98°, normal duration 89 milliseconds, T-wave upright from V_1_, giant T-wave V_2_, normal QTc interval, and no significant ST-segment change (Figure 1). Continuous telemetry (and 3-lead ambulatory Holter ECG) initially demonstrated occasional ventricular ectopy but no recurrent malignant arrhythmia.

**Figure 2:**
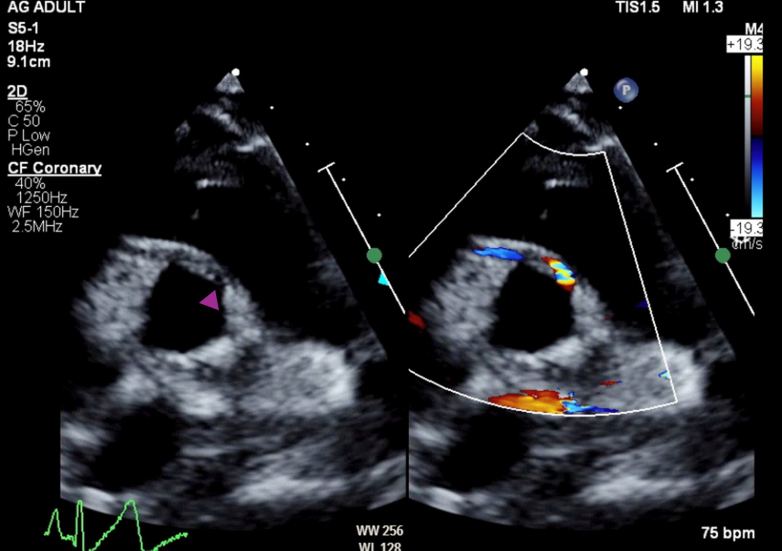
Initial Transthoracic Echocardiogram Transthoracic echocardiography, parasternal short-axis view, simultaneous 2-dimensional and color imaging, anomalous origin of the right coronary artery from the left-facing coronary sinus (purple arrowhead). The aortic origin of the left main coronary artery left main coronary artery was unable to be clearly seen.

Transthoracic echocardiography demonstrated an anomalous origin of the right coronary artery (RCA) from the left-facing coronary sinus. The aortic origin of the left main coronary artery (LMCA) was unable to be clearly seen (Figure 2). Biventricular function was preserved, with slight dyskinesis of the interventricular septum and overall slightly thickened myocardium.

**Figure 3:**
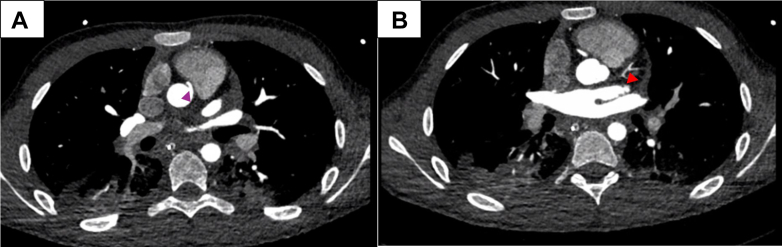
Initial Computed Tomography Scan Computed tomography scan (A) demonstrating the anomalous origin of the right coronary artery from the left facing coronary sinus, with an intra-arterial course between the aorta and pulmonary artery (purple arrowhead). (B) Left main coronary artery was suspected to be congenitally aplastic, with a hypoplastic left anterior descending coronary artery (red arrowhead).

The child proceeded to coronary computed tomography angiography, which confirmed the anomalous origin of the RCA from the left facing coronary sinus, with an interarterial course between the aorta and pulmonary artery, with no clear intramural course seen (Figure 3A, Supplementary Image 1, Video 1). The LMCA was suspected to be congenitally aplastic, with a hypoplastic left anterior descending coronary artery (Figure 3B) likely reconstituted via small perforator vessels. The circumflex artery was not seen.

**Figure 4:**
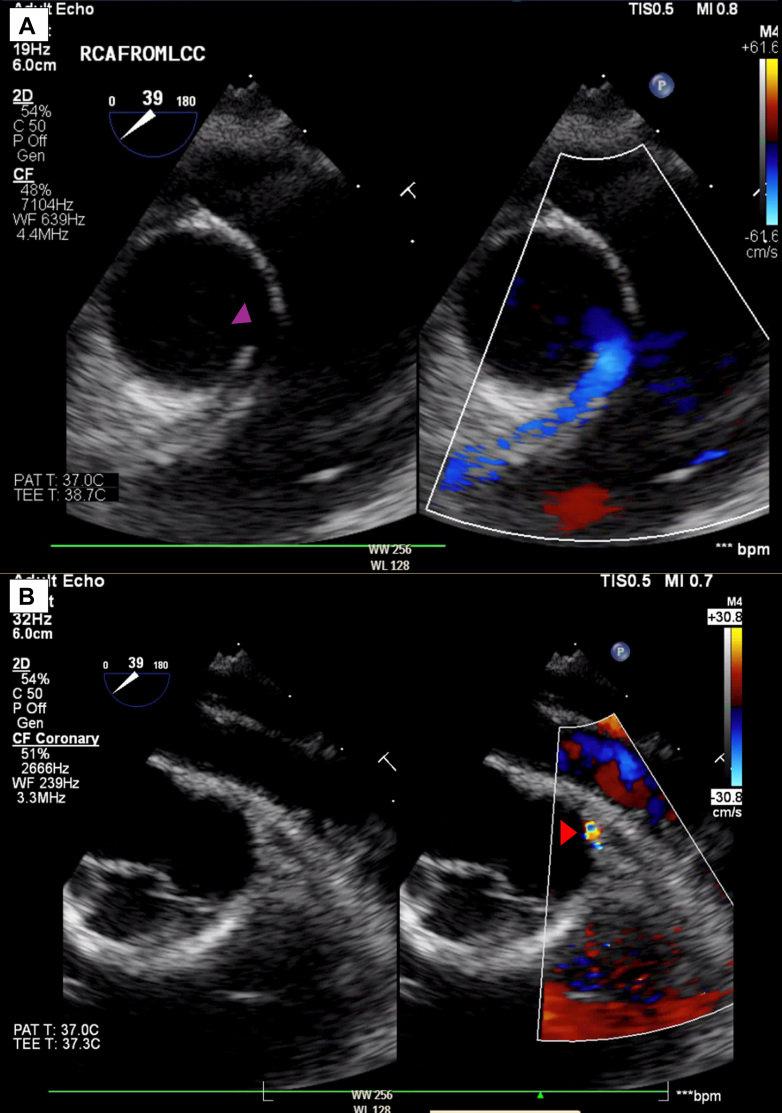
Initial Transesophageal Echocardiogram Transesophageal echocardiogram demonstrating (A) anomalous origin of the right coronary artery and (B) possible blind-ended coronary ostia at the typical left main coronary artery site.

**Figure 5:**
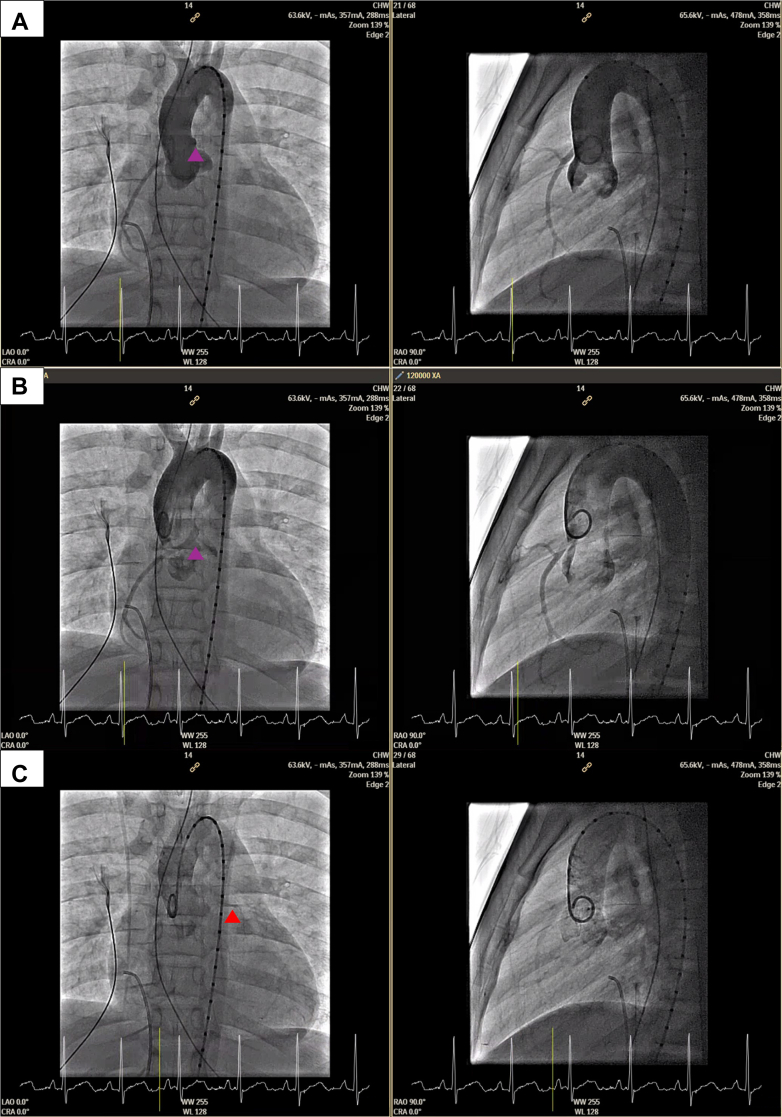
Initial Cardiac Catheter Imaging Paired anterior-posterior and lateral projections of aortic root angiogram using vessel sizing pigtail. Images shown in chronologic series: (A) early imaging demonstrates anomalous origin of the right coronary artery (purple arrowhead), (B) again shown later with less opacified aortic root, and finally (C) late retrograde filling of the left coronary artery system (red arrowhead).

A decision was made to proceed with transesophageal echocardiogram and invasive cardiac catheterization under general anesthesia. The transesophageal echocardiogram showed similar findings (Figure 4), with a possible blind-ended coronary ostia at the typical site of the LMCA. Aortic root angiogram confirmed the dominant RCA with extensive small collaterals perfusing the left coronary system, but no antegrade flow to the LMCA, which was late filling (Figure 5). Pulmonary artery angiogram did not identify any pulmonary artery origin of the coronary arteries.

The initial differential diagnosis of an acute occlusive coronary artery event was considered but thought to be less likely in the absence of typical ECG changes, preserved cardiac function, and absence of evidence of ongoing ischemia.

**Figure 6:**
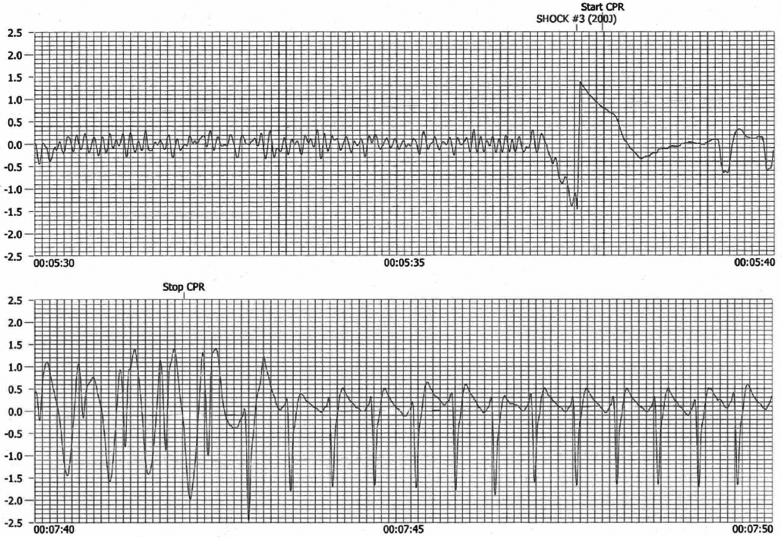
Automated External Defibrillator Trace Automated external defibrillator trace demonstrating ventricular fibrillation followed by presumptive sinus conducted rhythm with ST-segment change.

The automated external defibrillator recording was obtained; it showed 3 shocks for ventricular fibrillation, with the third 200-J shock resulting in sinus rhythm with ST-segment change (Figure 6).

The child did not show overt signs of postanoxic encephalopathy. Neuroimaging was reassuring. Standard routine post-cardiac arrest neuroprotective measures were applied in the intensive care unit. The child proceeded to extubation without any obvious neurologic insult identified following.

## Management

After multidisciplinary review, the child underwent surgical intervention via a median sternotomy with cardiopulmonary bypass. A decision was made to address both coronary artery anomalies. Operative findings included a small left anterior descending coronary artery, ostial atresia of the LMCA, and anomalous origin of the RCA. The left internal mammary artery (LIMA) was anastomosed to the left anterior descending artery (LAD) to provide antegrade left coronary artery system perfusion. The anomalous RCA was unroofed. Intraoperative echocardiography confirmed satisfactory graft patency and improved coronary perfusion. After the procedure, intraoperative transesophageal echocardiography demonstrated unobstructed flow through the LIMA graft.

## Outcome and Follow-Up

The early postoperative repair was complicated by a small pericardial effusion, which was managed conservatively. To further assess myocardial viability and confirm the absence of infarction, cardiac magnetic resonance was performed, which demonstrated no myocardial fibrosis. Repeat coronary computed tomography angiography scan demonstrated antegrade flow in the RCA and flow to the left system via the LIMA graft (Supplementary Image 2, Video 2). The child was discharged 4 weeks after presentation on empirical beta blockade, exercise restriction, and without an implantable cardioverter-defibrillator, after extensive discussion with the family and among the treating team.

**Figure 7:**
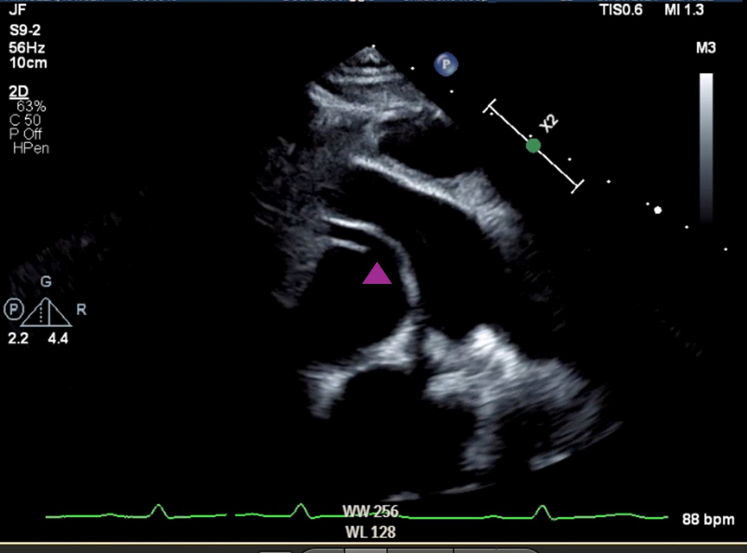
Follow-Up Transthoracic Echocardiogram Transthoracic echocardiogram 2 months after repair, parasternal short-axis view, showing unroofed right coronary artery (purple arrowhead).

**Figure 8:**
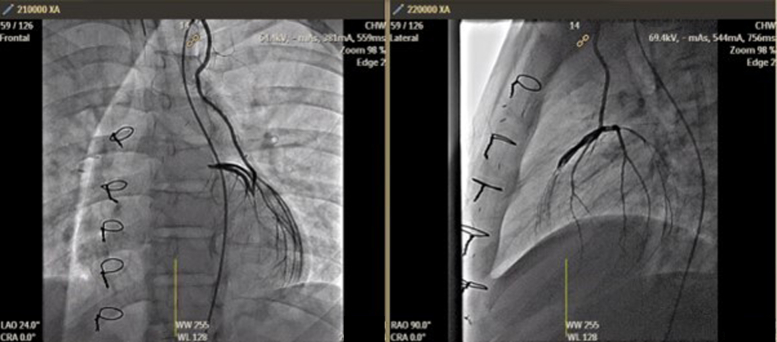
Repeat Cardiac Catheterization Repeat cardiac catheterization 3 months after surgical repair; selective angiogram of the left internal mammary artery (LIMA) demonstrating patency of the left coronary artery system via LIMA graft.

In early follow-up, the child remained well with no cardiac symptoms. Serial echocardiography confirmed patent left and RCA systems with flow evident on color Doppler (Figure 7). Biventricular systolic function was preserved, with no regional wall motion abnormalities at rest. Repeat cardiac catheterization at 3 months confirmed patent graft and improved left coronary perfusion (Figure 8). Exercise stress test (treadmill) did not identify any definite evidence of coronary artery ischemia. Exercise echocardiography (reclining bicycle) did not identify any regional wall motion abnormalities.

At 6-month follow-up, the child remained asymptomatic including during gradual return to sport. Long-term monitoring is planned to assess graft patency and potential late complications.

## Discussion

Left main coronary artery atresia (LMCAA) is an incredibly rare congenital anomaly characterized by the absence of a direct connection of the LMCA to the aortic root.^2^ Anomalous origin of the RCA, although more common, may be an incidental finding in otherwise asymptomatic individuals. Certain high-risk features or presentation with symptoms are thought to confer higher risk.^3^ The concurrent presence of an atretic LMCA and anomalous RCA with high-risk features is thought to be exceedingly rare.^4^

In LMCAA, myocardial perfusion relies entirely on collateral vessels arising from the RCA.^5^ During exertion, myocardial demand may outstrip supply, leading to ischemia-induced ventricular arrhythmias. The presentation varies with age; infants may experience heart failure, whereas adolescents and adults are more likely to present with exertional syncope or ischemia-related arrhythmias.^6^ Sudden cardiac death was reported as the presenting symptom in some cases.^7^ In the case of this child, it is postulated that the concurrent RCA anomaly with some high-risk features may have been contributory. In addition to the potential insufficiency of collateralization, the anomalous RCA may have been subject to exertional compromise, noting its acutely angled origin and interarterial course.^2^ Stress imaging was deemed unsafe in the postarrest period in the presence of known coronary artery anomalies. A decision was made to definitively embark on both unroofing of the RCA and LIMA to LAD coronary artery bypass grafting (CABG) to address both possible contributors.

Diagnosis of congenital coronary artery abnormalities, including LMCAA, requires a multimodal imaging approach. Although echocardiography can demonstrate abnormal coronary flow patterns, cardiac computed tomography angiography and coronary angiography remain the gold standards for defining coronary anatomy.^8^ These imaging modalities are critical in differentiating LMCAA from other congenital coronary anomalies and planning surgical intervention.^6^ Coronary artery abnormalities are typically sporadic, with rare syndromic associations. However, the presence of some familial clustering raises the possibility of a potential genetic contribution; this is an area of future research.

Surgical revascularization is the definitive treatment for LMCAA and symptomatic or high-risk anomalous origin of the RCA, with the approach tailored to the patient's anatomy. CABG using the LIMA to the LAD artery is widely regarded as the preferred option because of its superior long-term patency.^9^ Alsalehi et al^10^ noted a shift after 2005 toward coronary reconstruction osteoplasty over CABG for pediatric left main coronary artery ostial atresia, because it restores physiological antegrade flow and avoids competitive flow issues. In younger children with small internal mammary arteries, osteoplasty might be favored over CABG. However, in patients of near adult size with reasonable sized internal mammary arteries and good-sized coronary targets, CABG is a straightforward option. Long-term follow-up with stress imaging and serial coronary evaluations is essential because late complications such as graft stenosis or progressive coronary disease may develop.

The present case highlights the importance of early recognition and intervention in LMCAA, emphasizing the role of multimodal imaging and surgical expertise in optimizing patient outcomes.

## Conclusions

Congenital coronary anomalies should be considered in pediatric patients presenting with exertional syncope or cardiac arrest. Multimodal imaging plays a crucial role in diagnosis and surgical planning. This case highlights the importance of early recognition and timely intervention to optimize clinical outcomes.

Long-term follow-up is essential for these patients because late complications such as graft stenosis, progressive coronary disease, and arrhythmias may emerge. Continued surveillance with stress imaging and serial cardiac evaluations is recommended.

## Funding Support and Author Disclosures

The authors have reported that they have no relationships relevant to the contents of this paper to disclose.

## Appendix

Video 1 Preintervention Coronary Computed Tomography Angiography The right coronary artery arises from the medial aspect of the ascending aorta, just superior to the sinotubular junction. The proximal right coronary artery has an interarterial course between the aorta and the pulmonary trunk, as it turns to cross toward the right side of the heart. There is a relatively acute angle of the origin of the right coronary artery. No intramural segment is seen. There are no focal stenoses. The proximal left main coronary artery is not seen, and no normal ostium is demonstrated. There is reconstitution of a vessel at the expected location of the left anterior descending artery, presumably secondary to small perforator vessels. The circumflex artery is not identified, possibly absent.

Video 2 Postintervention Coronary Computed Tomography Angiography Right coronary artery: origin is superior to the sinotubular junction with normal caliber of proximal, mid, and distal part with no narrowing. Left coronary artery: origin is not clearly seen but mid and distal part is opacified. Graft anastomosis of the left internal mammary artery is noted with the anastomotic site seen distally.

Supplemental Figures Supplemental Figure 1: Pre-Interventional Three-Dimensional CT Reconstruction of Coronary Arteries Supplemental Figure 2: Post-Interventional Three-Dimensional CT Reconstruction of Coronary Arteries
