# Supplementary material for: Coexisting Congenital Left and Right Coronary Artery Anomalies: A Rare Cause of Pediatric Cardiac Arrest
Source: JACC Case Rep. 2025 Dec 8;31(2):105290. doi: 10.1016/j.jaccas.2025.105290 (PMC12925948; doi:10.1016/j.jaccas.2025.105290)
Supplement: Supplemental Figures — Supplemental Figure 1: Pre-Interventional Three-Dimensional CT Reconstruction of Coronary Arteries Supplemental Figure 2: Post-Interventional Three-Dimensional CT Reconstruction of Coronary Arteries [file mmc3.docx]

Supplemental Figure 1 PRE 3D


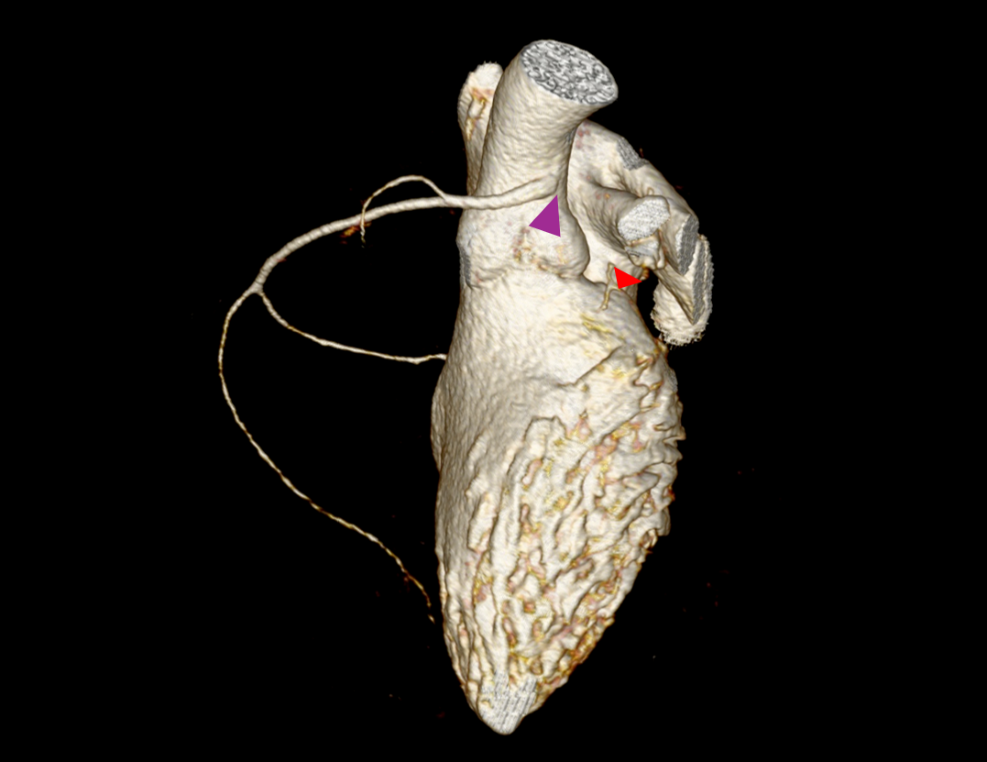


Pre-interventional 3D volume-rendered reconstruction of the coronary tree showing anomalous origin of Right Coronary Artery (purple arrow) and Left coronary artery stump (red arrow)

Supplemental Figure 2 POST 3D


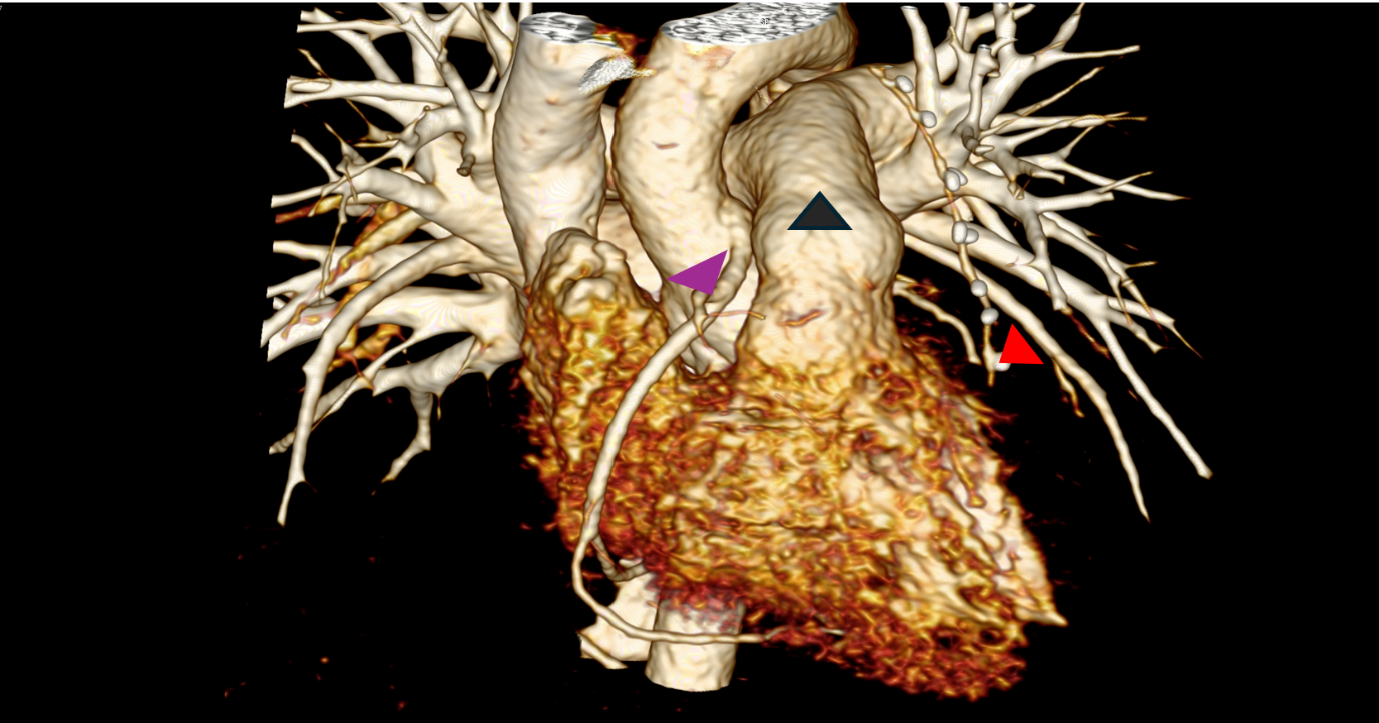


Post-interventional 3D volume-rendered reconstruction of the coronary tree showing an unroofed right coronary artery (purple arrow) and left internal mammary artery graft with clips(artefacts) along it connecting to the left coronary artery system (red arrow). RVOT has also been labelled (black arrow)
